# Changes in long-term life expectancy and years of life lost following the Great East Japan Earthquake in Fukushima Prefecture

**DOI:** 10.1038/s41598-025-88513-3

**Published:** 2025-02-14

**Authors:** Makoto Kosaka, Hiroaki Saito, Michio Murakami, Kyoko Ono, Yuka Ikeda, Akihiko Ozaki, Masaharu Tsubokura

**Affiliations:** 1 Department of Radiation Health Management, Fukushima Medical University School of Medicine, 1 Hikarigaoka, Fukushima, 960-1295 Fukushima Japan; 2 Orange Home-Care Clinic, Fukui, Fukui Japan; 3 Imamura General Hospital, Kagoshima, Kagoshima Japan; 4 Department of Internal Medicine, Soma Central Hospital, Soma, Fukushima Japan; 5 Department of Health Risk Communication, Fukushima Medical University School of Medicine, Fukushima, Fukushima Japan; 6 Center for Infectious Disease Education and Research, Osaka University, Suita, Osaka Japan; 7 Research Institute of Science for Safety and Sustainability, National Institute of Advanced Industrial Science and Technology Tsukuba West, Tsukuba, Ibaraki Japan; 8 Department of Radiation Disaster Medicine, Fukushima Medical University School of Medicine, Fukushima, Fukushima Japan; 9 Department of Breast and Thyroid Surgery, Jyoban Hospital of Tokiwa Foundation, Iwaki, Fukushima Japan

**Keywords:** Natural disaster, Life expectancy, Evacuation, Public health, Epidemiology

## Abstract

Prolonged evacuation after disasters increases the risks of diseases and challenges in healthcare access. This study aimed to analyze changes in life expectancy (LE) and years of life lost (YLL) in Fukushima Prefecture after the Great East Japan Earthquake. LE and YLL were calculated based on a dataset of 276,314 deaths in Fukushima Prefecture from January 2006 to December 2018 and were aggregated and compared in three time periods: 2006–2010, 2012–2015, and 2016–2018. LE and YLL were obtained using a life table method, focusing on four major causes of death: heart diseases, cerebrovascular diseases, pneumonia, and cancers. The overall LE for both males and females in Fukushima prefecture showed an increasing trend in all three periods. Notably, in 2012–2015, the increase in LE and YLL due to cancer was greater in areas with evacuation zones compared to those without evacuation zones. In contrast, in 2016–2018, a notable decrease in YLL due to cerebrovascular diseases for both males and females and due to heart diseases for males was observed in areas with evacuation zones compared to areas without. LE continued to rise in Fukushima Prefecture despite the significant long-term impact of the disaster on the population. Post-disaster changes in LE and YLL differed between areas with and without evacuation zones, suggesting that implementation of effective measures and policies in the region contributed to a successful recovery. This study may be crucial for evaluating future health measures and conditions in Fukushima Prefecture.

**Supplementary Information:**

The online version contains supplementary material available at 10.1038/s41598-025-88513-3.

## Introduction

Disasters and pandemics exert both direct and indirect effects on the overall health of a population. Large-scale, complex disasters impose long-term evacuation of residents, and several intertwined factors, such as the capacity of medical institutions, governmental responses to the disaster or pandemic, changes in the living environment, and alterations in disaster-related risk perception, lead to public health challenges^1,2^. A comprehensive analysis of various disaster-related phenomena and their mitigation strategies, considering both positive and negative health effects, is essential. Although disasters are mainly associated with adverse consequences, the resilience concept in disaster management emphasizes the need to focus on positive aspects as well^3^. A relevant indicator for a comprehensive, long-term assessment of the health impacts of a disaster on the population is years of life lost (YLL). This indicator is also used to calculate the global burden of disease, alongside measures such as disability-adjusted life years^4^. Analyzing local health indicators based on these measures helps to understand the current situation and identify areas that require attention.

The Great East Japan Earthquake in March 2011 had a significant impact on the eastern coast of the Tohoku region, with Fukushima Prefecture particularly affected by the Fukushima Daiichi nuclear power plant accident. This incident led to mandatory and voluntary evacuations in some areas, causing substantial changes in the resident’s lives and local medical landscape. As of February 2012, approximately 62,000 evacuees had relocated outside the prefecture, and approximately 96,000 had evacuated within the prefecture. The number of evacuees gradually decreased as evacuation orders were progressively lifted based on predictions of annual radiation exposure levels. By February 2018, the number of evacuees had declined to approximately 34,000 outside the prefecture and approximately 16,000 within the prefecture. Despite studies indicating that external and internal radiation exposure in the region had minimal health effects^5^, the long-term evacuation and changes in medical care and lifestyle have resulted in various health implications^6^. There have been reports of increased risks of diseases like diabetes, hypertension, dyslipidemia, and psychiatric disorders^7,8^. Furthermore, delayed cancer treatment due to social isolation resulting from the evacuation^9^ and reduced participation in cancer screening for several years after the disaster have been observed^10,11^. The large-scale evacuation also raised the demand for healthcare services despite the lack of adequate facilities and healthcare resources^12^.

Thus, prolonged evacuation is recognized as a significant factor adversely affecting the health of residents, particularly in the affected areas such as Hama-dori, the coastal area of Fukushima prefecture. However, some reports suggest an improvement in certain health indicators in areas that required long-term evacuation, such as Soma and Minamisoma cities, where residents were compelled to evacuate due to the tsunami and nuclear power plant accidents. In these two cities, a decrease in YLL due to heart disease in females and cerebrovascular disease in both males and females was observed in 2011–2015 compared to pre-disaster levels^13^. It is hypothesized that these improvements resulted from a synergistic effect of several factors, including the introduction of disaster-related health measures, an increase in health consciousness among the population, and advancement in local medical care. Nevertheless, there is a dearth of reports on these health indicators in other regions. Fukushima Prefecture exhibits diversity in terms of evacuation status, local population, economic conditions, and medical resources. Hence, comprehensively capturing long-term changes in life expectancy (LE) and YLL across Fukushima Prefecture is crucial for analyzing the health effects and status of the local population post-disaster and for devising appropriate countermeasures.

This study investigated the changes in LE and YLL over a 13-year period, both before and after the Great East Japan Earthquake, by analyzing mortality data from various regions of Fukushima Prefecture. We aimed to understand the long-term health effects of evacuation orders, local medical resources, and economic conditions on LE and YLL. The study’s findings will provide valuable data for assessing the impact of the disaster and evaluating the effectiveness of measures implemented to address the health issues of the local population.

## Methods

### Definition and calculation of LE and YLL

The main indicators in this study were LE and YLL due to the main causes of death. LE is one of the population health indicators, and YLL is defined as the difference between LE under specific risk and LE in a risk-free situation. The calculations and definitions in this study followed those in a previous report^13^. Briefly, LE was calculated using data from a population life table comprising the number of survivors and deaths, age-specific mortality rates, and the overall survival time of the population. Using these parameters, we could calculate LE at a given age $\:x$ by drawing a survival curve for the population (Supplementary Fig. 1). We drew one survival curve without a specific cause of death and another survival curve considering all causes of death. The difference between the integral values of these curves was the YLL.

### Vital statistics and population data

We obtained mortality data from the vital statistics and population data, which encompassed the vital registration data for Fukushima prefecture from January 2006 to December 2018. Although the data were not publicly available, the study was retrospective in nature, thereby informed consent was waived by the approval committee.　For every reported death within this dataset, we obtained the following data items: sex, age at death, cause of death (categorized using the International Classification of Diseases and Health-Related Problems, 10th Revision), the municipality where the deceased was registered at the time of death, and the exact time of death. To distinctly analyze the change before and after the disaster, we excluded the data for 2011. Consequently, our dataset comprised 276,314 recorded deaths: 107,827 for 2006–2010 (before), 94,770 for 2012–2015 (early), and 73,717 for 2016–2018 (late) periods. The corresponding proportions of females in these periods were 47.0%, 48.9%, and 49.9%. To create a survival curve, we calculated the mortality rate for each age group (0–100 + years). The mortality rate at age $$\:x$$$$\:\left({q}_{x}\right)$$ was calculated by dividing the number of deaths at age $$\:x\:\left({d}_{x}\right)$$ by the number of surviving people at age $$\:x\:\left({l}_{x}\right)$$. Survival curves were generated separately for males and females. Population data for each district were based on the Basic Resident Register, a nationwide resident registry network maintained by municipal units (city/town/village) using aggregate data as of March 31 of each year, which includes foreign residents. The Basic Resident Register and vital statistics are compiled and managed by the government and represent the most reliable official data on the Japanese population. However, it should be noted that these data are disaggregated based on where individuals report/register as living. After the Great East Japan Earthquake, many individuals remained registered at their original address, even if they had been evacuated for extended periods. Identifying the actual locations of these individuals is challenging. In the present study, as in previous studies, population and death data were calculated based on the residence information provided in these official statistics.

### District units in Fukushima Prefecture

**Fig. 1:**
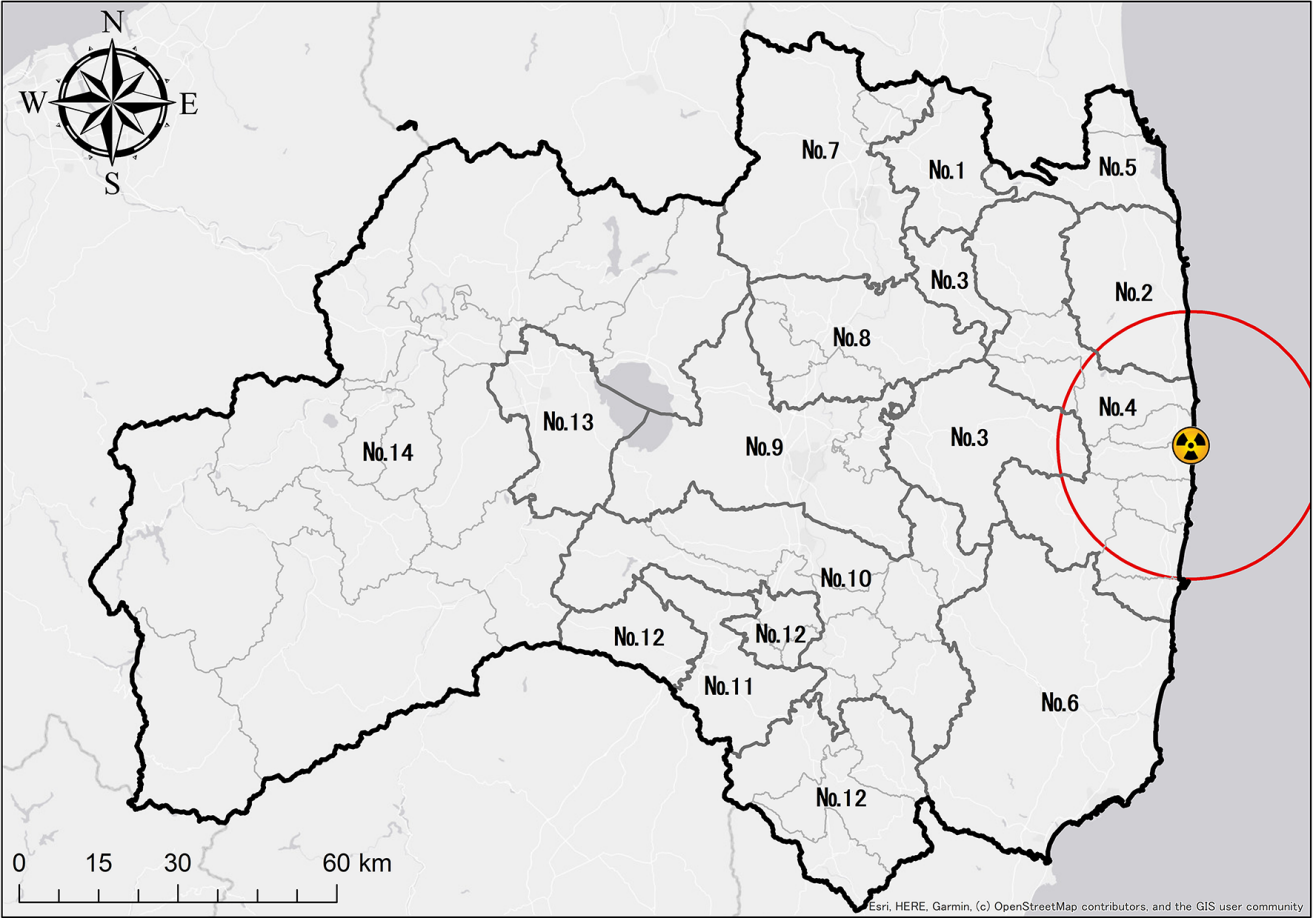
Fourteen districts in Fukushima Prefecture. We integrated neighbouring cities and towns into fourteen districts. Population data for each of the fourteen districts were combined and averaged for each of the three periods (2006–2010, 2012–2015, 2016–2018) to calculate mortality rates.

Mortality sub-data are reported from residences in units of 59 cities, towns, and villages. Since it is necessary to have the similar size of population for the calculation of LE and YLL to some extent, the data were consolidated into 14 districts according to regional characteristics and population sizes (Fig. 1, Supplementary material).

### Characteristics of the 14 districts

Data for these municipalities were collected from the statistical yearbook published by the Fukushima Prefecture. The characteristics in 2010, before the disaster, were used as the background information for each municipality. Population density, annual household income, number of medical personnel per 1,000 population (doctors, dentists, pharmacists), number of medical facilities per 100,000 population, percentage of primary industry, percentage of population over 65 years old, natural growth rate, and social growth rate for each municipality were weight averaged by the local population and calculated for each of the 14 districts. Municipal financial indicators were taken as their averages. In addition, we included whether the 14 districts included evacuation order areas at the time of the 2011 earthquake. Evacuation order zones were established after the Great East Japan Earthquake^14^. Based on this classification, districts 2, 3, and 4 were set as districts that included evacuation order zones.

### Calculation of mortality and YLL due to four major causes of death

Calculation of mortality rates and YLL due to specific causes was based on a previous report^13^. For the subject area, mortality rates were calculated as 4- to 5-year averages (i.e., 2006–2010, 2012–2015, 2016–2018) based on the data shown in Table 1.

**Table 1:** Age- and sex-specific counts of death in the pre- and post-disaster period in Fukushima Prefecture.

	2006–2010		2012–2015		2016–2018	
Age at death, yr	Male	Female	Male	Female	Male	Female
0–9	182	154	127	79	67	64
10–19	125	56	96	39	63	37
20–29	411	180	280	95	155	59
30–39	729	333	432	200	305	149
40–49	1487	719	931	499	686	365
50–59	4509	1910	2616	1191	1710	796
60–69	7894	3378	6795	2913	5366	2240
70–79	16,545	9232	11,601	6558	8277	4415
80–89	19,204	20,347	19,129	18,730	14,088	14,008
90–99	5849	13,457	6186	14,823	5985	13,507
100-	192	934	211	1239	227	1148
Total Population	1,016,045 (in 2006)	1,074,062 (in 2006)	963,776 (in 2012)	1,019,215 (in 2012)	945,024 (in 2016)	966,909 (in 2016)

The mortality rate was calculated for Fukushima Prefecture as a whole or for each of the 14 districts by sex. For ages 1–94 years, the mortality rate was calculated using a modified version of the method described by the Ministry of Health Labour and Welfare (MHLW), and for ages 0 and more than 95 years, the mortality rate was estimated using the method and parameters described by the MHLW^15,16^. LE was obtained by life table analysis using age-specific mortality rates. The YLL was obtained at ages 0, 40, and 65 years. We analyzed the following four major causes of death: heart diseases (ICD10: I05- 09,20–25,30–52), cerebrovascular disease (I60- 69), pneumonia (J12-18), and all cancers (C00 -97).

### Measurement of effect size

We measured the effect sizes for each of the explanatory variables (population density, income, number of medical personnel, number of medical institutions, percentage of primary industry, aging rate, financial capability index, natural population change, social population change rate, and whether evacuation zones are included) for the pre-disaster period and early post-disaster period. To quantify the effects of changes in LE and YLL in the pre-disaster, early post-disaster, and late post-disaster periods, we measured the effect sizes for each variable. The effect size was considered significant when the absolute value of the correlation coefficient exceeded 0.5^17^.

### LE and YLL sensitivity analysis

In addition to calculating the point estimates for LE and YLL, we estimated the uncertainty interval (UI) following the approach described in a previous report^13^. Given the annual variations observed in both population and mortality rates in the subject area, we assumed a normal distribution for these variations. Using the Monte Carlo simulation with Crystal Ball software (Oracle, Redwood City, CA, USA), we generated random numbers based on the 3 to 5-year averages (2006–2010, 2012–2015, 2016–2018) and standard deviation for both populations and crude mortality rates at ages 0–94 years. We employed the Latin hypercube method for sampling, setting the number of trials to 10,000. Distinct sets of random numbers were generated for all causes of death combined and for each specific cause of death individually. Subsequently, we calculated the YLL for each trial. The calculations were performed for Fukushima Prefecture as a whole in a consolidated manner and independently for each of the 14 districts. The results for Fukushima Prefecture as a whole are presented as the median and 2.5th to 97.5th percentile interval (95% UI) based on the Monte Carlo simulations. For the 14 districts, the median from the Monte Carlo simulation was used as the point estimate, and the standard error of the point estimate for the 14 districts was calculated to provide a confidence interval (95% CI) for comparisons between non-evacuation and evacuation areas. In addition to LE and YLL for age 0 years, calculations for ages 40 and 65 years were also performed as a sensitivity analysis.

The approval for this study was obtained from the Ethics Committees of Fukushima Medical University (Approval No. 30272). Informed consent was waived in accordance with Ethical Guidelines for Medical and Health Research Involving Human Subjects. This research was performed in accordance with relevant guidelines/regulations, and the Declaration of Helsinki.

## Results

### Pre-disaster and post-disaster changes in LE and YLL

Changes in LE and YLL due to diseases in the entire Fukushima Prefecture are presented in Table 1. Over the periods 2006–2010, 2012–2015, and 2016–2018, the LE for males were 78.57 (95% UI: 78.20–78.95), 79.49 (95% UI: 79.14–79.84), and 80.12 (95% UI: 79.76–80.49) years, respectively. As for females, the corresponding LEs were 85.51 (95% UI: 85.20–85.81), 86.02 (95% UI: 85.77–86.28), and 86.29 (95% UI: 85.05–86.54) years. Compared to the 2006–2010 period, the difference in LE for males increased significantly in the 2012–2015 period by 0.93 years (95% UI: 0.41–1.45) and 2016–2018 period by 1.55 years (95% UI: 1.04–2.08) (Supplementary Table 1). Additionally, the difference in LE for females showed a significant increase in the 2012–2015 period by 0.51 years (95% UI: 0.12–0.91) and in the 2016–2018 period by 0.78 years (95% UI: 0.39–1.16). The LE at ages 40 and 65 years showed a general increasing trend, although not statistically significant.

Among the four diseases, cancer accounted for the largest YLL for both males and females across all time periods, followed by heart diseases, cerebrovascular disease, and pneumonia. YLL due to cancer remained stable over the three periods (2006–2010, 2012–2015, and 2016–2018), with values of 3.72 (95% UI: 3.18–4.27), 3.59 (95% UI: 3.12–4.06), and 3.57 (95% UI: 3.09–4.06) years for males and 2.72 (95% UI: 2.31–3.13), 2.65 (95% UI: 2.31–2.99), and 2.69 (95% UI: 2.38–3.00) years for females, respectively. In the same periods, YLLs due to cerebrovascular disease, heart disease, and pneumonia did not worsen for both males and females, as shown in Table 2.

**Table 2:** Life expectancy and years of life lost in Fukushima Prefecture.

	Male			Female		
	2006–2010	2012–2015	2016–2018	2006–2010	2012–2015	2016–2018
Life expectancy at birth (years)	78.57(78.19–78.95)	79.49(79.13–79.85)	80.12(79.76–80.49)	85.51(85.21–85.81)	86.02(85.77–86.28)	86.29(86.04–86.54)
Years of Life Lost due to cancer (years)	3.72(3.18–4.27)	3.59(3.12–4.06)	3.57(3.09–4.06)	2.72(2.31–3.13)	2.65(2.31–2.99)	2.69(2.38–3.00)
Years of Life Lost due to cerebrovascular disease (years)	1.09(0.53–1.65)	0.90(0.40–1.40)	0.82(0.31–1.35)	1.07(0.64–1.49)	0.83(0.45–1.19)	0.76(0.41–1.10)
Years of Life Lost due to heart disease (years)	1.76(1.21–2.30)	1.74(1.26–2.23)	1.61(1.11–2.11)	1.49(1.08–1.91)	1.42(1.04–1.77)	1.22(0.89–1.57)
Years of Life Lost due to pneumonia (years)	0.78(0.25–1.33)	0.73(0.22–1.23)	0.59(0.07–1.10)	0.56(0.14–0.99)	0.48(0.11–0.84)	0.36(0.01–0.71)

Values are expressed as means and 95% uncertainty intervals for each district.

Supplementary Tables 1 and 2 show that a similar trend was observed regarding YLL at ages 40 and 65 years.

### Comparison of LE and YLL between evacuation and non-evacuation areas

The LE and YLL due to the four diseases in areas with and without mandatory evacuation are shown in Supplementary Fig. 2. The mandatory evacuation area had smaller pre-disaster YLLs due to cancer (3.45 years vs. 3.75 years) but larger pre-disaster YLLs due to cerebrovascular (1.30 years vs. 1.08 years) and heart diseases (1.88 years vs. 1.73 years) compared to those in non-evacuation areas (Supplementary Table 3). However, the mandatory evacuation area showed a decrease in post-disaster YLLs due to cerebrovascular and heart diseases, as shown in Supplementary Fig. 2. A similar trend was observed for YLL at ages 40 and 65 years, as shown in Supplementary Table 2.

### Effect sizes of variables on YLL changes

In examining the association between district characteristics and changes in LE relative to the 2006–2010 period for the four major diseases, several factors exhibited significant effect sizes, indicating their correlation with increases or decreases in LE. During the 2012–2015 period, population density (-0.623), number of medical personnel (0.550), and presence of an evacuation-designated zone (0.615, with ‘1’ denoting an area under evacuation, indicating a positive correlation) had notable effect sizes for male LE. In contrast, for female LE, per capita income showed a considerable effect size of 0.559 (Table 3).

**Table 3:** Effect sizes of regional characteristics on life expectancy calculated by Pearson’s correlation relative to the pre-disaster level.

	Changes in Life expectancy at birth relative to pre-disaster level			
	Males		Females	
Characteristics of the districts^†^	In 2012–2015	In 2016–2018	In 2012–2015	In 2016–2018
Population density	-0.623*	-0.542*	-0.206	0.121
Income per capita	0.064	0.313	0.559*	0.089
Number of medical professionals	-0.550*	-0.369	0.005	0.212
Number of medical institutions	-0.388	-0.201	0.176	0.385
Percentage of primary industry	0.390	0.247	-0.137	-0.131
Percentage of older population ( ≧ 65 year)	0.319	0.312	-0.141	0.124
Financial capability index	-0.023	0.067	0.376	-0.121
Natural population growth rate	-0.427	-0.349	0.104	-0.016
Net migration rate	-0.105	0.070	0.510	0.413
Including evacuation-ordered areas^‡^	0.615*	0.458	0.531	0.002

*P value < 0.05.

†Population density was calculated as the number of inhabitants per square kilometer; Per capita income is the annual per capita income (yen); Number of medical professionals is the sum of physicians, dentists, and pharmacists per 100,000 persons; Number of medical institutions was defined as a number of hospitals and clinics per 100,000 people; Financial capability index indicates the fiscal capacity of a local public entity, and is the average value obtained by dividing the standard fiscal revenue amount by the standard fiscal demand amount over the past three years; Natural population growth rate is the difference between the death rate and birth rate; Net migration rate is the difference between immigration and emigration; Whether or not the area includes areas under evacuation orders due to the 2011 disaster.

‡’1’ denoting an area under evacuation, indicating a positive correlation.

Regarding YLL, the effect sizes related to whether an area had an evacuation-designated zone were larger for males in the 2012–2015 period (0.580 for cancer) and in the 2016–2018 period (0.833 for cerebrovascular disease and 0.535 for heart diseases). For females in the 2016–2018 period, the effect size was 0.535 for cerebrovascular disease (Table 4).

**Table 4:** Change in life expectancy and years of life lost relative to pre-disaster levels and effect sizes.

	Male				Female			
	Area with evacuation	Area without evacuation	P value	Effect size (r) ^‡^	Area with evacuation	Area without evacuation	P value	Effect size (r)
Δ Life expectancy at birth (years), mean, SD ^†^								
2012–2015	1.64 (0.458)	0.88 (0.422)	0.02^*^	0.615	1.12 (0.946)	0.37 (0.402)	0.05	0.531
2016–2018	2.01 (0.696)	1.49 (0.382)	0.10	0.458	0.69 (0.134)	0.69 (0.381)	1.00	0.002
ΔYLL due to cancer at birth (years), mean, SD^§^								
2012–2015	0.16 (0.185)	-0.16 (0.203)	0.03^*^	0.580	-0.14 (0.155)	-0.02 (0.172)	0.28	-0.308
2016–2018	0.02 (0.334)	-0.11(0.174)	0.36	0.264	0.24 (0.174)	0.02 (0.313)	0.27	0.316
ΔYLL due to pneumonia at birth (years), mean, SD								
2012–2015	-0.08 (0.225)	-0.06 (0.092)	0.80	-0.073	-0.04 (0.134)	-0.06 (0.089)	0.68	0.120
2016–2018	-0.02 (0.324)	-0.22 (0.102)	0.08	0.482	-0.20 (0.061)	-0.18 (0.119)	0.74	-0.096
ΔYLL due to cerebrovascular disease at birth (years), mean, SD								
2012–2015	-0.27 (0.122)	-0.18 (0.145)	0.37	-0.261	-0.24 (0.103)	-0.23(0.063)	0.87	-0.049
2016–2018	-0.65 (0.097)	-0.25 (0.120)	< 0.01^*^	-0.833	-0.44 (0.111)	-0.30(0.093)	0.049^*^	-0.535
ΔYLL due to heart disease at birth (years), mean, SD								
2012–2015	-0.21 (0.189)	0.05 (0.190)	0.05	-0.528	-0.30 (0.358)	-0.06(0.152)	0.097	-0.461
2016–2018	-0.36 (0.177)	-0.08 (0.199)	0.049^*^	-0.535	-0.32 (0.233)	-0.25(0.261)	0.70	-0.114

*Statistically significant (*P* < 0.05).

† The change from the pre-disaster (2006–2010) period was calculated. The mean and standard deviation (SD) for each district are listed.

‡ Effect sizes were calculated by Pearson’s correlation.

§YLL; Years of life lost.

The same trend was observed as for LE and YLLs at age of 40 and 65 years as shown in Supplementary Table 4.

## Discussion

We examined the changes in LE and YLLs due to major diseases in Fukushima Prefecture after the 2011 Great Japan Earthquake, tsunami, and nuclear accident. Overall, the results indicated an increase in LE for both male and female populations in the entire prefecture during the post-disaster period. The increase in LE was even more significant in districts with mandatory evacuation areas. The rise in LE in Fukushima Prefecture, despite the significant long-term impact of the disaster, may be attributed to a combination of various factors, including the implementation of effective measures, policies that contributed to a successful recovery, and advances in medical technology. Regarding YLLs due to major diseases, the trends varied for each disease, and understanding the specific details of these changes is crucial for evaluating future health measures and conditions in Fukushima Prefecture.

The trends in YLL due to different diseases showed notable variations between districts with and without mandatory evacuation areas in Fukushima Prefecture. Overall, a decline was observed in YLLs due to pneumonia, cerebrovascular disease, and heart disease after the disaster. However, in districts with mandatory evacuation areas, there was a particularly significant decrease in YLL due to cerebrovascular disease for both males and females and heart diseases for males, compared to those in non-evacuation districts. Previous studies have indicated a correlation between natural disasters and the onset of ischemic heart disease^18^. Also, natural disasters have been reported to be associated with worsening of lifestyle diseases. A study reported worsening control of diabetes after Hurricane Katrina^19^. Other studies observed hypertension management issues due to evacuation after the disaster^8^ and increased hyperlipidemia after the disaster with and without evacuation^20^. On the contrary, in this study, YLLs due to these lifestyle diseases, particularly cerebrovascular and heart diseases, improved in the long term after the disaster in evacuated areas compared to those in non-evacuated areas. One possible explanation is that the districts with mandatory evacuation areas had larger pre-disaster YLLs due to heart and cerebrovascular diseases than those without mandatory evacuation areas, and thus the post-disaster improvements were more significant. The coastal locations where vascular diseases were more prevalent were designated as evacuation areas. In other words, this may reflect a process of post-disaster reduction in local medical and health inequalities that existed prior to the disaster due to limited medical resources. This hypothesis is supported by the observation that the increase in LE is negatively correlated with factors typically associated with health promotion, such as the number of physicians and medical facilities. The enhanced medical infrastructure, educational initiatives, and increased health awareness efforts during the reconstruction phase post-disaster may have contributed to the observed changes in YLL in the 2016–2018 period. Thus, despite the long-term adverse health effects of the disaster, medical and health disparities narrowed. However, these results need to be interpreted carefully, as the absolute YLL remains high, necessitating continued efforts to address lifestyle diseases and align medical services with healthcare needs.

In contrast to the declining YLL due to heart and cerebrovascular diseases and pneumonia, districts with mandatory evacuation areas showed a significantly larger YLL due to cancer after the earthquake than those without mandatory evacuation areas. Specifically, for males, districts with mandatory evacuation areas exhibited a significantly larger YLL due to cancer during the 2012–2015 period compared to districts without mandatory evacuation areas. Sensitivity analyses further confirmed that evacuation areas showed a substantially higher YLL due to cancer for males, both at ages 40 and 65 years, during this period compared to non-evacuation areas. Of note, these results are unlikely to be a direct effect of external radiation exposure from the Daiichi nuclear power plant, as shown in a previous study^21^, and the exact underlying factors are difficult to ascertain. However, it is possible that YLL due to cancer may have increased because medical care and health in the region improved post-disaster. A study projecting global YLL due to each disease in 2040 estimated an increase in YLL due to malignant tumors because of improved global public health conditions and medical standards^22^. Moreover, the increase in YLL due to cancer may be due to the impact of transient neglect of cancer prevention efforts after the disaster, as reported in Minamisoma City, including evacuated areas, where breast and colorectal cancer screening participation rates declined^10^. These effects might have manifested early after the disaster. Notably, the analysis of cancer screening patterns showed that males were less likely to receive cancer screening compared to females^10^, possibly explaining the sex difference in the post-disaster change in YLL due to cancer observed in this study.

In this study, we observed an overall increase in LE after the earthquake in the entire Fukushima prefecture. Individuals aged 65 years and above had a rising trend in LE even before the disaster. Notably, the study revealed that the post-disaster increase in LE was more prominent in districts with mandatory evacuation areas, with an effect size greater than 0.5 for both male and female populations during the 2012–2015 period. In contrast, major disasters, such as the Hunan floods in China, have been reported to cause a decrease in LE in the affected regions^23^. Similarly, during the COVID-19 pandemic, many countries experienced a decline in LE due to prolonged outbreaks and increased strain on healthcare facilities^24^. The LE changes depend on factors such as the scale of the disaster, timing and duration of health impacts, and resilience of healthcare institutions. In Fukushima Prefecture, there were concerns about adverse health effects, particularly in areas subjected to long-term evacuation, but the overall LE increased after the disaster. Of note, potential post-disaster survivorship bias should be considered when discussing changes in LE. Survivorship bias is a type of selection bias in which the population observed after a particular risk event, such as a disaster, underestimates the event’s impact because it comprises a risk-tolerant group that survived the event^25^. First, it is possible that older and frailer individuals were more likely to have been evacuated outside the current study area. However, this is unlikely, as older and frail individuals generally have more difficulty migrating to new environments. Indeed, publicly available data from the Fukushima Prefecture Disaster Response Headquarters suggest that a higher proportion of evacuees outside Fukushima Prefecture were younger individuals^26^. For instance, the number of evacuees outside the prefecture in May 2012 was approximately 60,000, whereas the number of evacuees under the age of 18 years in October 2012 was approximately 17,000. It is assumed that an equivalent number of individuals from the parental generation evacuated with them. Therefore, the impact of out-of-prefecture evacuation on the older and frail population was probably not statistically significant. In contrast, it is plausible that a significant number of frail older individuals passed away within the first year following the disaster, which may have contributed to the observed increase in LE among the remaining older population. However, it is unlikely that survivor bias alone was responsible for the changes observed in this study because it cannot explain the deterioration in YLL in the 2016–2018 period or the different trends of YLL depending on the disease. Additionally, the similar trend in YLL for individuals aged 40 and 65 years further supports the validity of the findings.

The increase in LE might be attributed to the narrowing of the medical care gap between the evacuated and non-evacuated districts. This is supported by the post-disaster decrease in YLLs due to heart diseases, cerebrovascular disease, and pneumonia, along with the increase in YLL due to cancer in the evacuated districts. These diseases, which tend to increase with age, may reflect improvements in the standards of medical care and public health for older adults. Given these results, it is likely that successful measures were taken during the disaster-recovery period, such as health measures by medical institutions, support from national and local governments, and heightened public health awareness. Following the disaster in Fukushima, health surveys and measures have been conducted more frequently. Fukushima Prefecture has started the Fukushima Health Management Survey, by commissioning Fukushima Medical University, and has shown a long-term commitment to the region’s health^27^. This ongoing initiative includes regular thyroid ultrasounds for youth, consistent medical checks in evacuated zones, mental health monitoring^28^, and surveys of pregnant women and infants. Crucially, it extends beyond assessment to establish enduring support systems. Additionally, a medical recovery plan was developed, which included restructuring the healthcare environment to accommodate the return of residents, constructing new medical facilities, and dispatching physicians in collaboration with universities. Other measures included waiving out-of-pocket medical expenses for disaster-affected individuals. Moreover, community-based initiatives, such as those involving community housing projects^29^, have also been undertaken. Although the health benefits of these initiatives should be carefully assessed, they may have contributed to improvements in LE and YLL in the study area.

### Future implications

This study offers a unique perspective by evaluating LE and YLL as key indicators of the long-term health effects of the Great East Japan Earthquake and by examining the relationship between evacuation measures and YLL due to various diseases. A more comprehensive analysis of the factors influencing YLLs and LE in these regions will be valuable in identifying and addressing future public health concerns. Although this analysis was confined to the major causes of death, a more detailed examination of each cause is necessary. For instance, it will be important to analyze cancer deaths by type and to investigate heart disease deaths more thoroughly, specifically examining different types such as ischemic heart diseases and heart failure. Additionally, assessing the impact of other large-scale disasters using a similar methodology and comparing the findings can help interpret similarities and differences in health challenges faced during various disasters. This study is particularly distinctive in that it includes both the disaster-stricken and surrounding areas and utilizes both life years lost and LE as long-term evaluative indicators. Future comparisons with other large-scale disaster scenarios remain a vital area for research. Additionally, a future challenge will be to investigate whether changes such as the expansion of medical facilities and increase in the number of physicians truly contributed to the extension of LE.

### Limitations

This study has some limitations. First, the causes of death analyzed are primarily chronic diseases and are not solely attributable to post-disaster conditions. Although the most common causes of death are examined, changes in LE loss due to other causes of death remain unknown. Second, the classification of causes of death may vary among physicians issuing the death certificates, potentially leading to inconsistencies. Nonetheless, the data utilized represent the most reliable sources currently available. Third, it remains unclear how specific individual interventions contributed to the reduction in LE disparities.

## Conclusion

In Fukushima Prefecture after the disaster, an overall increase in LE was observed compared to pre-disaster level. Notably, YLLs due to cerebrovascular disease, heart diseases, and pneumonia decreased following the disaster. Moreover, districts with mandatory evacuation zones registered more significant improvements in LEs, as well as YLL due to cancer. Analyzing the underlying causes of these changes in LE and YLL will be crucial in addressing future public health challenges in Fukushima Prefecture.

## Electronic supplementary material

Below is the link to the electronic supplementary material.

Supplementary Material 1

Supplementary Material 2

Supplementary Material 3

## Data Availability

The data that support the findings of this study are available on request from the corresponding author, HS. The data are not publicly available due to the restrictions of the use of vital statistics data.
