# Correction: Changes in long-term life expectancy and years of life lost following the Great East Japan Earthquake in Fukushima Prefecture

**DOI:** 10.1038/s41598-025-22308-4

**Published:** 2025-10-09

**Authors:** Makoto Kosaka, Hiroaki Saito, Michio Murakami, Kyoko Ono, Yuka Ikeda, Akihiko Ozaki, Masaharu Tsubokura

**Affiliations:** 1 Department of Radiation Health Management, Fukushima Medical University School of Medicine, 1 Hikarigaoka, Fukushima, 960-1295 Fukushima Japan; 2 Orange Home-Care Clinic, Fukui, Fukui Japan; 3 Imamura General Hospital, Kagoshima, Kagoshima Japan; 4 Department of Internal Medicine, Soma Central Hospital, Soma, Fukushima Japan; 5 Department of Health Risk Communication, Fukushima Medical University School of Medicine, Fukushima, Fukushima Japan; 6 Center for Infectious Disease Education and Research, Osaka University, Suita, Osaka Japan; 7 Research Institute of Science for Safety and Sustainability, National Institute of Advanced Industrial Science and Technology Tsukuba West, Tsukuba, Ibaraki Japan; 8 Department of Radiation Disaster Medicine, Fukushima Medical University School of Medicine, Fukushima, Fukushima Japan; 9 Department of Breast and Thyroid Surgery, Jyoban Hospital of Tokiwa Foundation, Iwaki, Fukushima Japan

Correction to: *Scientific Reports* 10.1038/s41598-025-88513-3, published online 14 February 2025

In the original version of this Article a wrong version of Figure 1 was published. The original Figure 1 and accompanying legend appear below.

**Fig. 1:**
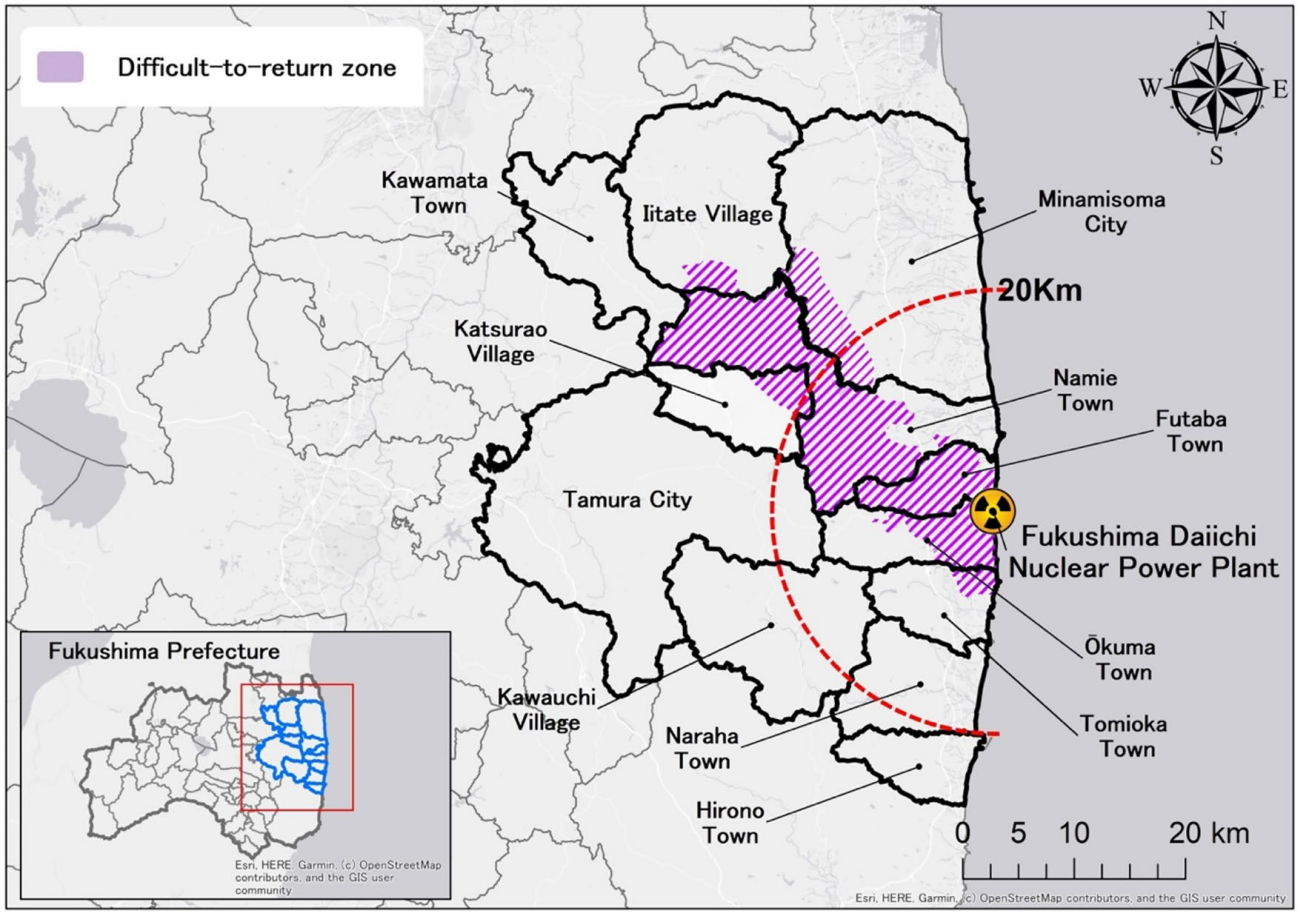
Cities and towns with evacuation orders in Fukushima prefecture. Population data for each of the 14 districts were combined and averaged for each of the three periods (2006–2010, 2012–2015, 2016–2018) to calculate mortality rates.

The original Article has been corrected.

