# Supplementary figures and images for: Changes in long-term life expectancy and years of life lost following the Great East Japan Earthquake in Fukushima Prefecture
Source: Sci Rep. 2025 Feb 14;15:5490. doi: 10.1038/s41598-025-88513-3 (PMC11828875; doi:10.1038/s41598-025-88513-3)

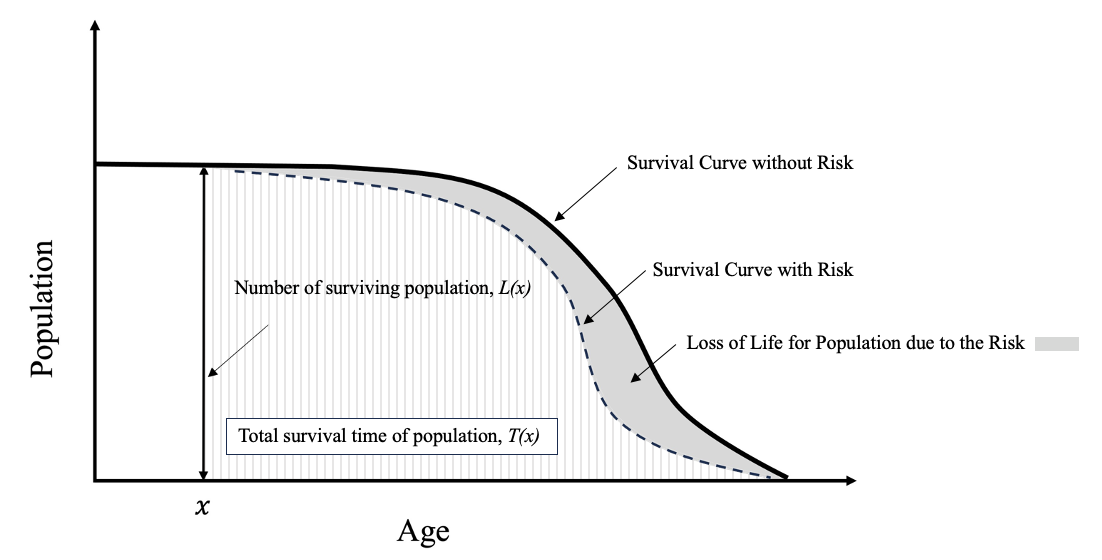

Supplement: Supplementary file 1 — Supplementary Material 1 [file 41598_2025_88513_MOESM1_ESM.png]

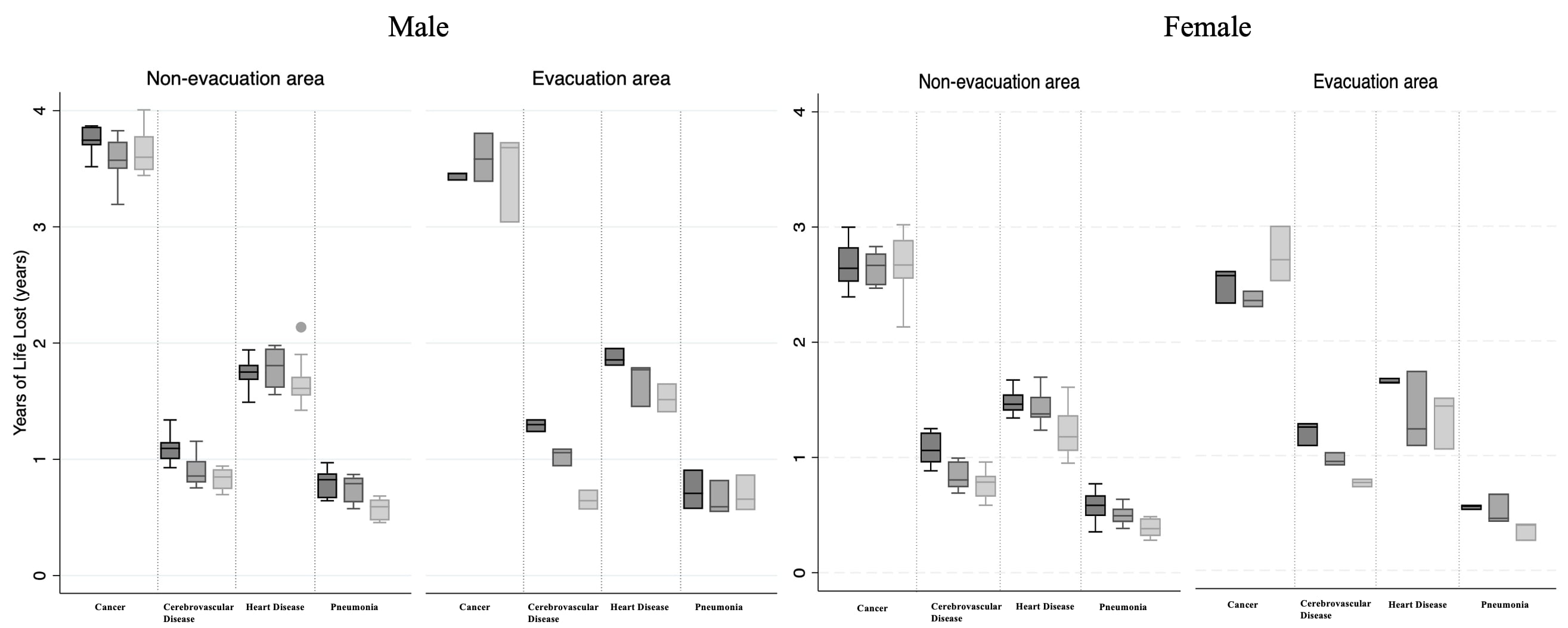

Supplement: Supplementary file 2 — Supplementary Material 2 [file 41598_2025_88513_MOESM2_ESM.jpg]
