# Supplementary material for: Changes in long-term life expectancy and years of life lost following the Great East Japan Earthquake in Fukushima Prefecture
Source: Sci Rep. 2025 Feb 14;15:5490. doi: 10.1038/s41598-025-88513-3 (PMC11828875; doi:10.1038/s41598-025-88513-3)
Supplement: Supplementary file 3 — Supplementary Material 3 [file 41598_2025_88513_MOESM3_ESM.docx]

**Supplementary material**

Supplementary Figure 1. **Conceptual diagram of survival curve and year of life lost**

Supplementary Figure 2. **Years of life lost (YLL) due to cancer, cerebrovascular disease, heart disease, and pneumonia**

Supplementary Table 1. **Changes in life expectancy and years of life lost in Fukushima Prefecture at Ages 40 and 65 years relative to pre-disaster levels**

Supplementary Table 2. **Life expectancy and years of life lost in Fukushima Prefecture at ages 40 and 65 years**

Supplementary Table 3. **Life expectancy and years of life lost in Fukushima Prefecture at age 0 years**

Supplementary Table 4. **Changes in life expectancy and years of life lost relative to pre-disaster levels and effect sizes depending on whether the area with or without evacuation area at ages 40 and 65 years**

**Breakdown of the 14 districts.**

1. Date City; 2. Minami-Soma City; 3. Kawamata, Tamura; 4. Kawauchi, Hirono, Iitate, Namie, Katsurao, Tomioka, Naraha, Futaba, Okuma; 5. Soma, Shinchi; 6. Iwaki City 7. Fukushima City, Koori, Kunimi 8.Nihonmatsu, Otamamura, Hongu 9. Koriyama, Miharu 10. Sukagawa City, Kagamiishi, Ten-ei, Ishikawa, Tamagawa, Hirata, Asakawa, Furudono, Ono, 11. Shirakawa, 12. Nishigo, Izumisaki, Nakajima, Yabuki, Tanagura, Yamatsuri, Hanawa, Samegawa, 13. Aizu Wakamatsu, 14. Kitakata City, Kitashiobara, Nishiaizu, Bandai, Inawashiro, Aizu Sakaoshita, Yukawa, Yagitsu, Mishima, Kanayama, Showa, Aizu Misato, Shimogo, Hinoemata, Tadami, Minami Aizu.

**Supplementary Table1: Changes in Life expectancy and years of life lost in Fukushima Prefecture at Age 40 and 65 years relative to pre-disaster levels.**

|  | **Male** |  | **Female** |  |
| --- | --- | --- | --- | --- |
| **ALL** | **2012–2015** | **2016–2018** | **2012–2015** | **2016–2018** |
| Life expectancy at birth (years) | 0·93(0·41–1·45) | 1·55(1·04–2·08) | 0·51(0·12–0·91) | 0·78(0·39–1·16) |
| Years of Life Lost due to cancer (years) | -0·12(-0·85–0·58) | -0·15(-0·88–0·56) | -0·07(-0·60–0·47) | -0·03(-0·55–0·50) |
| Years of Life Lost due to cerebrovascular disease (years) | -0·19(-0·94–0·56) | -0·27(-1·03–0·48) | -0·24(-0·81–0·32) | -0·32(-0·85–0·24) |
| Years of Life Lost due to heart disease (years) | -0·02(-0·75–0·71) | -0·14(-0·89–0·58) | -0·07(-0·63–0·46) | -0·26(-0·79–0·27) |
| Years of Life Lost due to pneumonia (years) | -0·06(-0·80–0·69) | -0·19(-0·91–0·56) | -0·08(-0·64–0·47) | -0·20(-0·74–0·34) |
|  |  |  |  |  |
| **Age 40 years** |  |  |  |  |
| Life expectancy at birth (years) | 0·87(0·37–1·38) | 1·36(0·86–1·87) | 0·38(0·01–0·77) | 0·72(0·34–1·09) |
| Years of Life Lost due to cancer (years) | -0·13(-0·81–0·55) | -0·18(-0·88–0·52) | -0·06(-0·59–0·45) | -0·03(-0·53–0·47) |
| Years of Life Lost due to cerebrovascular disease (years) | -0·19(-0·91–0·53) | -0·28(-1·01–0·45) | -0·24(-0·78–0·31) | -0·32(-0·84–0·22) |
| Years of Life Lost due to heart disease (years) | -0·02(-0·73–0·67) | -0·14(-0·84–0·57) | -0·07(-0·60–0·45) | -0·26(-0·77–0·26) |
| Years of Life Lost due to pneumonia (years) | -0·08(-0·81–0·64) | -0·19(-0·91–0·52) | -0·08(-0·63–0·44) | -0·19(-0·71–0·33) |
|  |  |  |  |  |
| **Age 65 years** |  |  |  |  |
| Life expectancy at birth (years) | 0·42(-0·03–0·86) | 0·79(0·32–1·25) | 0·22(-0·13–0·55) | 0·5(0·18–0·83) |
| Years of Life Lost due to cancer (years) | -0·06(-0·69–0·55) | -0·04(-0·68–0·6) | -0·03(-0·50–0·44) | 0·00(-0·44–0·45) |
| Years of Life Lost due to cerebrovascular disease (years) | -0·17(-0·81–0·48) | -0·27(-0·94–0·41) | -0·19(-0·68–0·30) | -0·29(-0·75–0·17) |
| Years of Life Lost due to heart disease (years) | -0·03(-0·65–0·60) | -0·09(-0·75–0·57) | -0·06(-0·54–0·41) | -0·22(-0·67–0·23) |
| Years of Life Lost due to pneumonia (years) | -0·09(-0·73–0·57) | -0·23(-0·9–0·45) | -0·09(-0·57–0·39) | -0·2(-0·66–0·27) |

**Supplementary table 2. Life expectancy and Years of life lost in Fukushima Prefecture at Age 40 and 65 years**

|  | **Male** |  |  | **Female** |  |  |
| --- | --- | --- | --- | --- | --- | --- |
| **Age 40 years** | **2006–2010** | **2012–2015** | **2016–2018** | **2006–2010** | **2012–2015** | **2016–2018** |
| Life expectancy at Age 40 years | 39·88(39·51–40·24) | 40·75(40·41–41·07) | 41·23(40·89–41·58) | 46·34(46·05–46·64) | 46·73(46·49–46·98) | 47·06(46·83–47·3) |
| Years of Life Lost due to cancer | 3·70(3·18–4·22) | 3·58(3·15–4·01) | 3·52(3·06–3·97) | 2·60(2·20–3·00) | 2·54(2·21–2·85) | 2·57(2·28–2·87) |
| Years of Life Lost due to cerebrovascular disease | 1·08(0·55–1·62) | 0·89(0·41–1·37) | 0·80(0·32–1·30) | 1·07(0·65–1·49) | 0·82(0·47–1·18) | 0·74(0·41–1·07) |
| Years of Life Lost due to heart disease | 1·70(1·16–2·22) | 1·66(1·21–2·13) | 1·55(1·08–2·03) | 1·46(1·06–1·87) | 1·39(1·03–1·73) | 1·20(0·88–1·53) |
| Years of Life Lost due to pneumonia | 0·78(0·25–1·31) | 0·71(0·23–1·20) | 0·59(0·09–1·09) | 0·55(0·14–0·97) | 0·47(0·12–0·82) | 0·36(0·02–0·69) |
| **Age 65 years** |  |  |  |  |  |  |
| Life expectancy at Age 65 years | 18·2(17·89–18·52) | 18·62(18·29–18·94) | 18·99(18·64–19·34) | 23·14(22·88–23·41) | 23·36(23·13–23·59) | 23·65(23·45–23·85) |
| Years of Life Lost due to cancer | 2·89(2·43–3·35) | 2·84(2·43–3·24) | 2·85(2·41–3·30) | 1·76(1·40–2·13) | 1·73(1·42–2·03) | 1·77(1·51–2·03) |
| Years of Life Lost due to cerebrovascular disease | 0·90(0·44–1·36) | 0·73(0·27–1·18) | 0·63(0·14–1·13) | 0·93(0·56–1·30) | 0·74(0·41–1·08) | 0·64(0·36–0·92) |
| Years of Life Lost due to heart disease | 1·30(0·84–1·76) | 1·26(0·83–1·70) | 1·21(0·73–1·69) | 1·34(0·99–1·69) | 1·28(0·95–1·60) | 1·12(0·84–1·40) |
| Years of Life Lost due to pneumonia (years) | 0·79(0·33–1·24) | 0·71(0·25–1·17) | 0·57(0·05–1·07) | 0·55(0·18–0·92) | 0·46(0·14–0·79) | 0·35(0·06–0·63) |

**Supplementary table 3 : Life expectancy and Years of life lost in Fukushima Prefecture at Age 0 depending on whether the area with or without evacuation area**

|  | **Male** |  | **Female** |  |
| --- | --- | --- | --- | --- |
|  | **Area with evacuation** | **Area without evacuation** | **Area with evacuation** | **Area without evacuation** |
| Life expectancy at birth (years), mean, 95%CI |  |  |  |  |
| 2006–2010 | 78·36 (77·03–79·69) | 78·58 (78·15–79) | 85·57 (84·89–86·26) | 85·57 (85·28–85·87) |
| 2012–2015 | 79·99 (79·76–80·23) | 79·46 (79·17–79·75) | 86·7 (84·84–88·55) | 85·94 (85·59–86·29) |
| 2016–2018 | 80·37 (79·07–81·66) | 80·06 (79·71–80·41) | 86·26 (85·9–86·62) | 86·26 (85·87–86·65) |
| YLL due to cancer at birth (years), mean, 95% CI |  |  |  |  |
| 2006–2010 | 3·45 (3·35–3·54) | 3·75 (3·66–3·84) | 2·50 (2·11–2·89) | 2·67 (2·55–2·78) |
| 2012–2015 | 3·61 (3·08–4·14) | 3·59 (3·46–3·71) | 2·36 (2·18–2·54) | 2·65 (2·56–2·74) |
| 2016–2018 | 3·47 (2·54–4·39) | 3·64 (3·52–3·75) | 2·74 (2·14–3·35) | 2·69 (2·51–2·86) |
| YLL due to pneumonia at birth (years), mean, 95% CI |  |  |  |  |
| 2006–2010 | 0·73 (0·32–1·15) | 0·80 (0·73–0·88) | 0·55 (0·49–0·61) | 0·56 (0·48–0·64) |
| 2012–2015 | 0·66 (0·27–1·04) | 0·75 (0·67–0·82) | 0·52 (0·18–0·85) | 0·50 (0·45–0·55) |
| 2016–2018 | 0·71 (0·31–1·11) | 0·58 (0·52–0·64) | 0·35 (0·14–0·56) | 0·39 (0·34–0·44) |
| YLL due to cerebrovascular disease at birth (years), mean, 95% CI |  |  |  |  |
| 2006–2010 | 1·30 (1·16–1·44) | 1·08 (0·99–1·17) | 1·20 (0·94–1·47) | 1·07 (0·98–1·16) |
| 2012–2015 | 1·03 (0·85–1·21) | 0·90 (0·83–0·98) | 0·96 (0·81–1·12) | 0·84 (0·77–0·91) |
| 2016–2018 | 0·65 (0·41–0·89) | 0·83 (0·76–0·89) | 0·76 (0·67–0·86) | 0·77 (0·70–0·84) |
| YLL due to heart disease at birth (years), mean, 95% CI |  |  |  |  |
| 2006–2010 | 1·88 (1·7–2·07) | 1·73 (1·64–1·82) | 1·65 (1·58–1·72) | 1·48 (1·41–1·55) |
| 2012–2015 | 1·67 (1·2–2·13) | 1·78 (1·66–1·91) | 1·35 (0·50–2·21) | 1·42 (1·32–1·53) |
| 2016–2018 | 1·53 (1·21–1·85) | 1·66 (1·51–1·80) | 1·33 (0·72–1·94) | 1·23 (1·08–1·38) |

CI, confidence interval; YLL, years of life lost

**Supplementary table 4. Changes in life expectancy and years of life lost relative to pre-disaster levels and effect sizes depending on whether the area with or without evacuation area at Age 40 and 65 years**

|  | **Male** |  |  |  | **Female** |  |  |  |
| --- | --- | --- | --- | --- | --- | --- | --- | --- |
|  | **Area with evacuation** | **Area without evacuation** | **P value** | **Effect size (r) ^‡^** | **Area with evacuation** | **Area without evacuation** | **P value** | **Effect size (r) ^‡^** |
| **Age 40 years** |  |  |  |  |  |  |  |  |
| ΔLife expectancy at birth (years), mean, SD |  |  |  |  |  |  |  |  |
| 2012–2015 | 1·29(0·303) | 0·85(0·362) | 0·080 | 0·483 | 1·11 (0·599) | 0·27 (0·240) | <0·01 | 0·751 |
| 2016–2018 | 1·76(0·500) | 1·35(0·350) | 0·130 | 0·427 | 0·79 (0·332) | 0·65 (0·390) | 0·59 | 0·159 |
| ΔYLL due to cancer at birth (years), mean, SD |  |  |  |  |  |  |  |  |
| 2012–2015 | 0·17(0·175) | -0·16(0·233) | 0·043 | 0·547 | -0·16 (0·023) | -0·03 (0·140) | 0·17 | 0·390 |
| 2016–2018 | 0·03(0·348) | -0·15(0·181) | 0·220 | 0·347 | 0·29 (0·247) | 0·01 (0·328) | 0·20 | 0·362 |
| ΔYLL due to pneumonia at birth (years), mean, SD |  |  |  |  |  |  |  |  |
| 2012–2015 | -0·10(0·223) | -0·07(0·114) | 0·750 | 0·095 | -0·09 (0·053) | -0·06 (0·084) | 0·64 | 0·138 |
| 2016–2018 | -0·06(0·275) | -0·22(0·116) | 0·140 | 0·416 | -0·20 (0·062) | -0·17 (0·097) | 0·59 | 0·159 |
| ΔYLL due to cerebrovascular disease at birth (years), mean, SD |  |  |  |  |  |  |  |  |
| 2012–2015 | -0·26(0·134) | -0·19(0·151) | 0·450 | 0·218 | -0·23 (0·109) | -0·23 (0·067) | 0·93 | 0·028 |
| 2016–2018 | -0·63(0·091) | -0·27(0·127) | <0·001 | 0·800 | -0·43 (0·122) | -0·31 (0·096) | 0·10 | 0·464 |
| ΔYLL due to heart disease at birth (years), mean, SD |  |  |  |  |  |  |  |  |
| 2012–2015 | -0·13(0·18) | 0·02(0·18) | 0·210 | 0·358 | -0·33 (0·259) | -0·16 (0·487) | 0·56 | 0·169 |
| 2016–2018 | -0·27(0·11) | -0·10(0·178) | 0·140 | 0·413 | -0·41 (0·178) | -0·24 (0·252) | 0·32 | 0·287 |
| **Age 65 years** |  |  |  |  |  |  |  |  |
| ΔLife expectancy at birth (years), mean, SD |  |  |  |  |  |  |  |  |
| 2012–2015 | 0·65(0·34) | 0·43(0·273) | 0·250 | 0·328 | 0·89(0·494) | 0·08(0·240) | <0·01 | 0·770 |
| 2016–2018 | 1·28(0·547) | 0·80(0·332) | 0·070 | 0·495 | 0·84(0·186) | 0·45(0·305) | 0·59 | 0·521 |
| ΔYLL due to cancer at birth (years), mean, SD |  |  |  |  |  |  |  |  |
| 2012–2015 | 0·18(0·086) | -0·11(0·186) | 0·020 | 0·604 | -0·08(0·15) | -0·02(0·123) | 0·17 | 0·200 |
| 2016–2018 | 0·06(0·221) | -0·03(0·12) | 0·330 | 0·279 | 0·22(0·092) | 0·00(0·218) | 0·20 | 0·435 |
| ΔYLL due to pneumonia at birth (years), mean, SD |  |  |  |  |  |  |  |  |
| 2012–2015 | -0·12(0·233) | -0·07(0·115) | 0·600 | 0·155 | -0·12(0·023) | -0·06(0·077) | 0·64 | 0·315 |
| 2016–2018 | -0·11(0·186) | -0·25(0·131) | 0·170 | 0·392 | -0·20(0·035) | -0·18(0·088) | 0·59 | 0·132 |
| ΔYLL due to cerebrovascular disease at birth (years), mean, SD |  |  |  |  |  |  |  |  |
| 2012–2015 | -0·22(0·023) | -0·18(0·122) | 0·640 | 0·139 | -0·15(0·074) | -0·18(0·079) | 0·93 | 0·187 |
| 2016–2018 | -0·50(0·05) | -0·27(0·101) | 0·003 | 0·730 | -0·38(0·080) | -0·29(0·095) | 0·10 | 0·416 |
| ΔYLL due to heart disease at birth (years), mean, SD |  |  |  |  |  |  |  |  |
| 2012–2015 | -0·07(0·264) | 0·00(0·115) | 0·470 | 0·210 | -0·29(0·275) | -0·01(0·137) | 0·56 | 0·592 |
| 2016–2018 | -0·18(0·185) | -0·05(0·128) | 0·200 | 0·368 | -0·42(0·157) | -0·21(0·185) | 0·32 | 0·446 |

Δ, change; SD, standard deviation; YLL, years of life lost
